# Profil clinique et bactériologique des infections néonatales bactériennes à l'Hôpital Laquintinie de Douala, Cameroun

**DOI:** 10.11604/pamj.2016.23.97.8523

**Published:** 2016-03-15

**Authors:** Sandrine Kemeze, Béatrice Moudze, Andreas Chiabi, Charlotte Eposse, Alexis Kaya, Madeleine Mbangue, Odette Guifo, Innocent Kago

**Affiliations:** 1 Institut Supérieure des Sciences de la Santé, Université des Montagnes, Bangangté, Cameroun; 2 Département de Pédiatrie, Hôpital Laquintinie de Douala, Cameroun; 3 Service de Pédiatrie et sous Spécialités Pédiatriques, Hôpital Gynéco-Obstétrique et Pédiatrique de Yaoundé, Cameroun; 4 Faculté de Médecine et des Sciences Biomédicales de Yaoundé, Cameroun; 5 Laboratoire d'Analyse Biomédical, Hôpital Laquintinie de Douala, Cameroun

**Keywords:** Infection néonatale bactérienne, clinique, écologie bactérienne, Cameroun, Neonatal bacterial infection, clinical, bacterial ecology, Cameroon

## Abstract

**Introduction:**

L'Organisation Mondiale de la Santé a estimé la survenue globale de décès néonatal à 2,8 millions en 2015, dont 47,6% étaient dues aux infections. Ces infections peuvent survenir chez un nouveau-né de 0 à un mois de vie, pouvant aller jusqu’à 3 mois.

**Méthodes:**

C'est une étude prospective allant du 1er mars au 30 juin 2015 au Service de néonatologie de l'Hôpital Laquintinie de Douala. Etaient inclus tout nouveau-né symptomatique avec ou sans critère anamnestique et tout nouveau-né asymptomatique, présentant au moins un risque infectieux et ayant au moins une culture positive ou une anomalie de la numération formule sanguine ou une protéine C réactive positive.

**Résultats:**

Des 310 nouveau-nés admis, 300 ont été retenus pour infection néonatale, soit une incidence de 96,8%. Nous avons réalisé 104 cultures dont 25 positives, soit une incidence de l'infection néonatale confirmée de 24%. Les facteurs associés à l'infection étaient la prématurité inexpliquée <35 semaines d'aménorrhée(45,1%) et la réanimation néonatale (34,8%). La fièvre (56%) et les troubles neurologiques (48,8%) étaient les manifestations cliniques les plus fréquentes. Les Gram négatifs étaient les germes les plus fréquents (56%). L'imipenème (95%) et l'amikacine (66,7%) étaient les antibiotiques les plusefficaces. L’évolution était favorable dans 66,4% des cas et le taux de décès était de 33,6%.

**Conclusion:**

Cette étude révèle une forte prévalence de l'infection dans cet hôpital. L’écologie bactérienne est dominée par les Gram négatifs, on note une importante résistance aux antibiotiques usuels et une mortalité assez élevée.

## Abstract (en)

**Introduction:**

The World Health Organization has estimated that the global incidence of neonatal deaths was 2,8 million in 2015, of which 47,6% were due to infections. These infections can affect newborns babies ages 0-1 month through 3 months.

**Methods:**

This is a prospective study conducted from 1 March to 30 June 2015 in the Neonatology service of the Laquintinie Hospital at Douala. All symptomatic newborns with or without anamnestic criteria and all asymptomatic newborns, with at least an infectious risk and a positive blood culture or an abnormal blood count or positive C-reactive protein were included in the study.

**Results:**

Of the 310 newborns enrolled in the study, 300 were retained for neonatal infection, corresponding to a total incidence of 96.8%. We performed 104 cultures, of which 25 were positive, corresponding to an incidence of confirmed neonatal infection of 24%. The factors associated with infection were unexplained preterm birth < 35 weeks of amenorrhea (45,1%) and neonatal resuscitation (34,8%). Fever (56%) and neurological disorders (48.8%) were the most frequent clinical symptoms. Gram-negative bacteria were the most frequent germs (56%). Imipenem (95%) and amikacin (66.7%) were the most effective antibiotics. Outcome was favorable in 66,4% of cases and the overall mortality rate was 33,6%.

**Conclusion:**

This study revealed a high prevalence of neonatal bacterial infection in this Hospital. Bacterial ecology was dominated by Gram-negative bacteria. It was recorded a significant resistance to the most widely used antibiotics and a fairly high mortality.

## Introduction

L'infection néonatale (INN) est un syndrome clinique de bactériémie caractérisé par des signes et symptômes cliniques survenant chez un nouveau-né de 0 à un mois de vie, pouvant s’étendre jusqu’à 3 mois [1, 2]. C'est un problème majeur de santé publique de par sa forte mortalité. En effet, l′Organisation Mondiale de la Santé (OMS) estime la survenue globale de décès néonatal à 2,8 millions en 2015 et 47,6% sont dues à l'INN [3]. Au Cameroun à l'Hôpital Laquintinie de Douala (HLD) en 2015, le taux de mortalité lié à l'INN était de 54,93% [4]. Il se poserait donc ici un problème de prise en charge adéquate de ces infections. Cette étude visait donc à déterminer le profil épidémiologique, clinique, bactériologique et évolutif de l'infection néonatale bactérienne à l'Hôpital Laquintinie de Douala.

## Méthodes

Nous avons mené une étude prospective au Service de néonatalogie de l'Hôpital Laquintinie de Douala du 1^er^ Mars 2015 au 30 Juin 2015 sur les nouveau-nés de 0 à 28 jours de vie. Étaient inclus dans notre étude les nouveau-nés répartis en deux groupes comme suit; Groupe A: Tous les nouveau-nés symptomatiques avec ou sans critères anamnestiques. Groupe B: Tous les nouveau-nés asymptomatiques et présentant au moins un risque infectieux, tout nouveau-né présentant une pathologie non-infectieuse. Le matériel utilisé était le matériel d'examen clinique, le matériel de prélèvement et d'analyse biologique, le matériel de secrétariat. Concernant la procédure de collecte des données, pour chaque nouveau-né reçu et inclus dans notre étude, nous avons procédé à un interrogatoire de la mère pour son identification puis nous avons utilisé le carnet de suivi de la grossesse de la mère et la fiche de transfert ou de référence du nouveau-né pour regrouper les éléments anamnestiques et les événements survenus pendant l'accouchement. Un examen physique détaillé (avec recueil de paramètres vitaux et anthropométriques) était fait à la recherche de signes en faveur d'une infection. Puis, nous procédions aux prélèvements bactériologiques: hémoculture, analyse du liquide céphalo-rachidien (LCR), examen cytobactériologique des urines (ECBU); biologiques (NFS et CRP) et parasitologique (hémoparasite). Une fois les prélèvements effectués et acheminés sans délais au laboratoire pour analyse, les nouveau-nés étaient mis sous antibiothérapie probabiliste (ampicilline, céfotaxime et aminoside). L'analyse statistique s'est faite grâce aux logiciels Epi info version 3.5.4 pour la saisie des données ainsi que le calcul des moyennes et des pourcentages et Microsoft Excel 2010 pour la reproduction graphique des résultats sous forme d'histogrammes et de tableaux.

## Résultats

Pendant notre période d’étude, 310 nouveau-nés ont été admis et 300 ont été retenu pour INN, soit une incidence de l'INN de 96,8%. Nous avons confirmé l'infection sur 25 prélèvements bactériologiques soit une incidence de l'INN confirmée de 24%. La plupart des nouveau-nés infectés soit 94% avaient été consulté dans la période néonatale précoce (dont 85% dans la période très précoce) et 6% dans la période néonatale tardive. L’âge moyen des nouveau-nés infectés était 2,5 +/- 4 jours avec des extrêmes de 1 et 28 jours. Les nouveau-nés de sexe masculin étaient les plus infecté dans 62% des cas, soit un sexe ratio de 1,63. Vingt-neuf pourcent (29%) des mères avaient un âge qui variait entre 25 et 29 ans, 50,7% étaient multipares, 62,3% avaient un niveau d'instruction du secondaire et 29% étaient ménagères. Concernant le statut matrimonial, 30,6% étaient célibataires, 33,1% étaient mariées et 30,3% vivait en union libre. Les nouveau-nés prématurés étaient les plus atteints par l'INN dans 61,7%. Les facteurs de risques de l'infection les plus retrouvés dans notre étude étaient la prématurité inexpliquée inférieure à 35 semaines d'aménorrhée (SA) (45%), la réanimation néonatale dans les conditions douteuses d'asepsie (34,8%) et la rupture prolongée des membranes (RPM) supérieure à 12h (32,7%). Les faibles poids de naissance étaient majoritairement atteints par l'infection (60,3%). Les troubles thermiques étaient les manifestations cliniques les plus fréquentes (63,8%), dominées par l'hyperthermie (56%). Les troubles neurologiques, les troubles respiratoires et les troubles de comportement suivaient avec un pourcentage respectif de 48,8%, 43,1% et 41,2% (Tableau 1). L’écologie bactérienne était dominée par les bacilles Gram négatifs (56%) avec 52,6% dans la période néonatale précoce et 66,7% dans la période néonatale tardive (Tableau 2). La septicémie était le tableau clinique le plus représenté dans la période néonatale précoce (88,2%) par contre dans la période néonatale tardive, on retrouvait de façon égale l'infection urinaire et la méningite dans 50% des cas (Tableau 3). Les antibiotiques les plus efficaces étaient l'imipénème (100%) et l'amikacine (60%) pour les Gram négatifs, l'imipénème (90,9%) et l'amikacine (70%) pour les Gram positifs et de façon globale, L'imipénème (95%) et l'amikacine (66,7%) (Tableau 4). Des 300 nouveau-nés infectés, 253 ont eu une évolution sous traitement médical parmi lesquels 66,4% ont retrouvé la guérison et 33,6% sont décédés.

**Tableau 1:** Répartition des nouveau-nés selon les manifestations cliniques

Signes cliniques			Effectif	Pourcentage
Troubles thermiques	Hyperthermie = 93 Hypothermie = 73	}	166	63,8
Troubles neurologiques	Trouble tonus = 110 Trouble RA = 104 Convulsion = 16	}	127	48,8
Troubles respiratoires	Détresse respiratoire	}	112	43,1
Troubles du comportements	Refus tétée = 76 Geignement = 42 Pleurs incessants = 19 Irritabilité = 15		107	41,2
Troubles cutanéo-muqueux	Cyanose = 57 Ictère = 21	}	76	29,2
Troubles digestifs	Vomissement = 5 Ballonnement abdominal = 5 Diarrhée = 2	}	10	3,8

**Tableau 2:** Répartition des germes isolés selon la période néonatale (N= 25)

Gram	Germes	Tranche d’âge(en jour) 0-7 N (%) 8-28 N (%)		Total (%)
	E coli	7 (36,8)	2 (33,3)	9 (36)
Bacille Gram négatif	Klebsiella	2 (10,5)	2 (33,3)	4 (16)
	Pseudomonas	1 (5,3)	0	1 (4)
**Total Gram négatif**		10 (52,6)	4 (66,7)	14 (56)
Cocci Gram positif	Staphylococcus aureus	9 (47,4)	2 (33,3)	11 (44)
**Total Gram positif**		9 (47,4)	2 (33,3)	11 (44)
**Total des germes**		19 (100)	6 (100)	25(100)

**Tableau 3:** Germes isolés selon la nature du prélèvement et en fonction de la période néonatale

Gram	Germes	ECBU	N=6(%)	LCR	N=2(%)	Hémoculture	N=17(%)
		Tranche d’âge en Jour 0-7 8-28 0-7			8-28	0-7	8-28
Gram négatif	E coli	01(16,7)	01(16,7)	01 (50)	0(0)	05 (29,4)	01(5,9)
	Klebsiella	01(16,7)	01(16,7)	0(0)	01 (50)	01 (5,9)	0(0)
	Pseudomonas	01(16,7)	0(0)	0(0)	0(0)	0(0)	0(0)
**Total Gram négatif**		03 (50)	02(33,3)	01 (50)	01(50)	06 (35,3)	01(5,9)
Gram positif	Staphylococcus aureus	0(0)	01(16,7)	0(0)	0(0)	09 (52,9)	01(5,9)
**Total Gram positif**		0(0)	01(16,7)	0(0)	0(0)	09 (52,9)	01(5,9)
**TOTAL**		03 (50)	03 (50)	01 (50)	01(50)	15 (88,2)	02(11,8)

**Tableau 4:** Répartition des germes selon leur pourcentage de sensibilité globale aux antibiotiques

Antibiotiques	Nombre de fois testé (Gram - et Gram+)	Nombre de fois sensible (Gram - et Gram+)	Sensibilité globale(%)
**BETALACTAMINES**			
Imipeneme	20	19	95
Cefotaxime	18	06	33,3
Meropeneme	10	03	30
Cefuroxime	08	02	25
Ampicilline	24	05	20,8
Ceftriaxone	19	03	15,8
Amoxi-clav	24	03	12,5
Ceftazidime	20	02	10
**AMINOSIDES**			
Amikacine	15	10	66,7
Gentamycine	21	07	33,3
Streptomycine	03	01	33,3
Tobramycine	22	04	18,2

## Discussion

L'incidence de l'INN bactérienne est de 96,8%. Ce résultat est supérieur à celui retrouvé par Chiabi et al. à l'Hôpital Gynéco-Obstétrique et Pédiatrique de Yaoundé (HGOPY) au Cameroun en 2011 (34,7%) [5] et largement supérieur à celui de Chemsi et al. en 2015 à Casablanca (6,2%) [6]. Cette fréquence hospitalière élevée peut s'expliquer par le fait que l'HLD est un hôpital de référence de niveau 2 et reçoit par conséquent les patients venant de toutes les formations sanitaires de la ville de Douala et des environs le plus souvent à des stades assez avancés de la pathologie. Ces nouveau-nés pour la plupart sont évacués par des moyens de transport le plus souvent non approprié et donc susceptibles d'acquérir les infections pendant ce transport. L'infection néonatale précoce a été la plus fréquente dans 94% des cas avec une nette prédominance dans la période néonatale très précoce (85%) soit les 72 premières heures de vie dans notre population d’étude. Ceci traduit le plus souvent une transmission verticale de la mère à l'enfant avant ou pendant l'accouchement [7]. Cette tendance de l'infection néonatale précoce fréquente est retrouvée à HGOPY en 2011 (87,6%) dans l’étude de Chiabi et al. [5]. Par contre, Vergano et al. au Nigéria en 2005 [2] avaient une prédominance de l'INN dans la période néonatale tardive. Ceci peut s'expliquer par les divergences dans la méthodologie. En effet, certains auteurs considèrent la période néonatale précoce comme étant celle qui va de 0 à 3 jours[1]. Dans notre étude, nous avons utilisé la définition de l'agence nationale d'accréditation et d’évaluation en santé (ANAES) qui considère la période néonatale précoce comme allant de 0-7 jrs. L'infection était fréquente chez les prématurés. Ce résultat corrobore à celui de Chiabi et al. en 2011 à HGOPY au Cameroun [5] et de Masson et al. en France en 2005 [8]. Cette observation pourrait s'expliquer par le fait que les prématurés sont faibles de par leur immaturité qui les expose plus que les autres à des infections. Par contre, Manta et al. en Inde en 2015 [9] ont noté une prédominance de l'infection chez les nouveau-nés à terme, ceci pourrait s'expliquer par le fait que leur population d’étude était dominée par les nouveau-nés à terme. La prématurité inexpliquée 35 SA, la réanimation néonatale dans des conditions douteuses d'asepsie et la rupture prolongée des membranes étaient les facteurs associés les plus retrouvés dans l'INN.

Au Cameroun, Chiabi et al. en 2011 à HGOPY [5] ont retrouvé la réanimation néonatale, la RPM et la fièvre péripartale comme facteurs associés les plus fréquents. En effet, la RPM, la fièvre péripartale et la prématurité inexpliquée < 35 SA sont des critères majeurs de l'INN définis par l'ANAES en 2002 [10]. La plupart des nouveau-nés infectés étaient de faible poids de naissance (< 2500g). Ces résultats sont similaires à ceux de Chiabi et al. à HGOPY [5] et de Manta et al. en Inde [9]. Ils sont différents de celui de Shah et al. au Népal [11] qui ont retrouvé une faible représentation de faible poids de naissance dans l'infection. Cette prédominance de l'infection chez les faibles poids de naissance dans notre étude pourrait s'expliquer par le fait que notre population d’étude était dominée par les petits poids de naissance, contrairement à celle de Shah et al. où la plupart des nouveau-nés avaient un poids de naissance ≥à 2500g. Les troubles de régulations thermiques dominés par la fièvre et les troubles neurologiques ont représenté les manifestations cliniques les plus fréquemment retrouvés dans notre série. Cette tendance est retrouvée par Chiabi et al. en 2011 à HGOPY[5] et en 2005 à Bertoua au Cameroun [12]. Manta et al. ont retrouvé en Inde en 2015 une prédominance de la léthargie et du refus de téter[9]. Nous avons eu 25 cultures bactériennes positives sur 104 faites dans notre étude, soit une incidence de positivité de l'INN de 24%. Le profil bactérien de façon globale dans notre série, a révélé une prédominance des bacilles Gram négatifs avec pour chef de file Escherichia coli (36%) suivie du Klebsiella pneumoniae (16%). Ce constat est similaire à celui de Chiabi et al. à Bertoua[12] au Cameroun. Il est également retrouvé dans d'autres pays comme le Maroc en 2015 [6], la Tunisie en 2013 [13] et l'Inde en 2015 [9]. Néanmoins nous avons eu 44% de Cocci gram positifs en occurrence le Staphylococcus aureus. Il était le germe le plus fréquent en l'Inde [14] et dans une étude menée par l'OMS en 2015 dans six pays en développement [15]. La forme clinique la plus fréquente a été la septicémie dans la période néonatale précoce (88,2%). Ce résultat corrobore à celui retrouvé par Chiabi et al en 2011 [5]. Nous avons retrouvé dans notre série, pendant la période néonatale tardive une répartition égale de l'infection urinaire et de la méningite dans 50% des cas. Jain et al. en 2003 au Nepal [1] retrouvait une prédominance de la méningite et Chiabi et al. à HGOPY en 2011 [5] avait une prédominance de l'infection urinaire dans la période néonatale tardive. Les Gram négatifs ont eu une bonne sensibilité à l'imipenème dans 100% des cas et à l'amikacine dans 60% des cas. Au Maroc, les grams négatifs étaient sensibles aux C3G (100%) et à l'ampicilline (48%) [6] tandis qu'en Inde, ils avaient une meilleure sensibilité à l'imipenème (92,8%), l'amikacine (77,2%), la céfotaxime (66,7%) et l'ampicilline (66,7%) [9]. Les Cocci Gram positifs quant à eux ont eu une bonne sensibilité à l'imipenème dans 90,9% des cas et à l'amikacine dans 70% des cas dans notre étude. L'imipenème testé dans cette étude était le Bacqure*. Au Maroc, l'ampicilline avait gardé une meilleure sensibilité sur les gram positifs [6]. En Inde, on a retrouvé une sensibilité des Cocci gram positif à la vancomycine (100%), la cefotaxime (100%), la gentamycine (86%) et la ciprofloxacine (66,7%) [14]. Des 300 nouveau-nés inclus dans notre étude, 253 ont eu une évolution sous traitement médical parmi lesquels 168 ont retrouvés la guérison et 85 sont décédés, soit un taux de décès de 33,6%. Notre taux de décès est semblable à celui d'Azoumah et al. au Togo (33%) [16], inférieur à celui de Kago et al. au Cameroun (46,7%) [17], supérieur à celui d'Akaffou et al. à Abidjan (26,7%) [18] et de Chiabi et al. à Bertoua au Cameroun (5,2%) [12]. Notre taux de décès peut s'expliquer par le retard de référence, les conditions de transport non appropriés des nouveau-nés et une mauvaise compliance au traitement par manque de moyens financiers.

## Conclusion

Au terme de notre étude, il en ressort que l'infection néonatale est la pathologie hospitalière la plus rencontrée dans la période néonatale. Sa présentation clinique est dominée par la fièvre, les troubles neurologiques et la détresse respiratoire. L’écologie bactérienne est dominée par les Gram négatif. L’évolution est favorable dans 66,4% des cas et le taux de décès néonatal dû à l'INN est de 33,6%, avec une prédominance dans la période néonatale précoce.

### Etat des connaissance sur le sujet

- L'infection néonatale survient chez les nouveau-nés de 0 à 28 jours.
- Les germes les plus fréquents sont les bacilles Gram négatifs.

### Contribution de notre étude a la connaissance

- L'infection néonatale prédomine dans la période néonatale précoce.
- La septicémie est le tableau clinique le plus fréquent.
- Les antibiotiques les plus efficaces sont l'imipénème et l'amikacine.
